# Effect of Air Drying on the Metabolic Profile of Fresh Wild and Artificial *Cordyceps sinensis*

**DOI:** 10.3390/foods13010048

**Published:** 2023-12-21

**Authors:** Tao Wang, Chuyu Tang, Mengjun Xiao, Zhengfei Cao, Min He, Jianzhao Qi, Yuling Li, Xiuzhang Li

**Affiliations:** 1 State Key Laboratory of Plateau Ecology and Agriculture, Qinghai University, Xining 810016, China; 13085500761@163.com (T.W.); chuyutang0410@163.com (C.T.); 15574237597@163.com (M.X.); c1474477969@163.com (Z.C.); hi-mi1228@163.com (M.H.); 2 College of Chemistry and Pharmacy, Northwest A&F University, Xianyang 712100, China; qjz@nwafu.edu.cn; 3 Qinghai Academy of Animal and Veterinary Science, Xining 810016, China

**Keywords:** *Cordyceps sinensis*, metabonomics, air drying, lysine biosynthesis

## Abstract

Fresh and dried *Cordyceps sinensis* are widely used by the public for medicinal and health purposes. However, the differences between them have not been examined. In this study, fresh wild and artificial *C. sinensis* (WFC and AFC) were dried to obtain dried wild and artificial *C. sinensis* (WDC and ADC). Non-targeted GC-MS was used to analyze the metabolic profile characteristics of the four groups of samples. The results showed that air drying significantly altered the composition and content of *C. sinensis*, mainly in the form of higher abundance of organic acids and derivatives and lower abundance of lipids and lipid-like molecules in fresh *C. sinensis*. Hierarchical cluster analysis (HCA) and quantitative analyses showed that air drying increased the abundance of Valine, Zinniol, Urocanate, Vulpinic acid, and Uridine 5’-diphosphate, and decreased Xanthotoxol, Vitexin-4-o-glucoside, Val-trp, and Wogonin. These differentially accumulated metabolites (DAMs) were also shown to be potential biomarkers for *C. sinensis*. KEGG enrichment analysis identified lysine biosynthesis as the most significantly enriched pathway. Annotation of these DAMs to lysine biosynthesis revealed that citrate cycle and pyruvate metabolism entered lysine biosynthesis via 2-oxohlutarate and Homocitrate, respectively, resulting in significant enrichment of L-saccharopine and L-lysine content was significantly higher. Alanine, aspartate, and Glutamate metabolism synthesized more L-aspartate to promote L-lysine synthesis. Thus, high levels of L-lysine result in lysine degradation and pymolysine, which are the most active metabolic pathways during the drying of fresh *C. sinensis* and indirectly lead to differences in metabolic profiles.

## 1. Introduction

*C. sinensis* is a rare traditional Chinese medicinal material. It has extremely high nutritional value and medicinal activity and is regarded as “medicine and food homology” by the National Health Commission of the People’s Republic of China [1]. It is mainly distributed in Tibet, Sichuan, Qinghai, Yunnan, and Gansu provinces in China, and grows in alpine meadow areas at 3, 500–5, 200 m. The extremely high altitude leads to the environment presenting low temperatures, low oxygen, variable temperatures, and high radiation and other climatic characteristics; in response to a variety of environmental factors, the metabolic profile of wild *C. sinensis* undergoes drastic changes. This also means that wild *C. sinensis* and artificial *C. sinensis* have different pharmacological functions [2,3]. The “Yuewang Yaozhen” and “Compendium of Materia Medica” recorded its sweet taste, which has the effects of tonifying kidneys and lungs, hemostasis, and resolving phlegm. Dry *C. sinensis* can be used to treat kidney deficiency and essence deficiency, impotence and spermatorrhea, lumbar and knee pain, chronic cough and asthma, and spleen and stomach diseases [4,5]. Modern pharmacological studies have shown that *C. sinensis* has many pharmacological effects, such as immune regulation, anti-tumor, anti-apoptosis, anti-oxidation, relieving renal failure, and treating cardiovascular diseases [6,7,8,9]. Its pharmacological effects are closely related to polysaccharides, nucleosides, sterols, flavonoids, cyclic peptides, phenols, anthracenes, polyketides, and alkaloids. A recent study showed that the water extract of *C. sinensis* had a significant anti-inflammatory effect on the infection of the compound influenza virus [10,11]. *C. sinensis* contains a water-insoluble polysaccharide, β-(1,3) glucan. As a component of the fungal cell wall, it is also the core structure of immunoactivity polysaccharides, which stimulates immune receptors such as Dectin-1 to trigger innate immune response and enhance individual immunity [12,13]. Cordycepin, a kind of adenosine, has been shown to inhibit the differentiation of T cells into regulatory T cells (Treg, a suppressive phenotype of T cells) and delay tumor growth in tumor-bearing mice [14,15]. According to the theory of traditional Chinese medicine, the medicinal function of *C. sinensis* lies in the combination of multiple metabolites. A single metabolite may also play an important role, but it cannot fully reflect the medicinal value of *C. sinensis*. Similarly, modern pharmacology and traditional Chinese medicine theory believe that the medicinal function of *C. sinensis* comes from a large number of special metabolites [16]. The difference is that traditional Chinese medicine tends to use dry *C. sinensis* and modern pharmacology chooses fresh *C. sinensis*. This results in confusion for ordinary consumers when choosing fresh or dried *C. sinensis* for physical health care.

The most common processing methods for *C. sinensis* are low-temperature freeze drying, high-temperature drying, and air drying. Studies have shown that preservation at 4 °C can keep *C. militaris* fresh for at least 12 months [17]. Unfortunately, they only analyzed the activity of fungi and the ability to produce fruiting bodies and did not point out the changes in metabolites. In recent years, the emerging vacuum freeze-drying method, in a vacuum low-temperature environment, has freeze dried *C. sinensis* quickly while maintaining the appearance, shape, and color of fresh products, and can well retain the protein type and content of *C. sinensis* and antioxidant activity, such as antioxidant dismutase [18,19]. However, it has high requirements for the operation of instruments and methods and cannot be widely used by consumers. On the contrary, hot air drying is not conducive to the retention of proteins, especially proteins with relative molecular weight of 25–100 kDa, which are prone to structural changes during drying and which directly leads to the loss of their functions [20]. Fortunately, this method retains higher sterols, such as ergosterol, cholesterol, and stigmasterol. The authors conjectured that the change in sterol content in *C. sinensis* under high-temperature treatments enhances cell membrane stability and helps to resist environmental changes [21,22]. The study also pointed out that dried *C. sinensis* products are resistant to storage, not easy to corrupt, have increased flavor, and are more suitable for cooking [23]. At present, hot air drying and steam dehydration drying are the most widely used methods. The advantage of this method is that it is not affected by external climate factors and is more suitable for large-scale continuous factory operations. The disadvantages are that the drying time is long, the loss of nutrients is significant, and the production energy consumption is large and thus does not meet the requirements of energy conservation and emission reduction.

What is encouraging is that the drying method of *C. sinensis* is also stipulated in the “Pharmacopoeia of the People’s Republic of China 2020” as air drying and drying at low temperatures [24]. What needs to be considered is whether and how the metabolites of *C. sinensis* will change after air drying and whether these metabolite changes will give fresh and air-dried *C. sinensis* different medicinal values. This is one of the urgent problems in the medicinal field of *C. sinensis*. Based on non-targeted metabolomics, wild and artificial fresh *C. sinensis* were air dried in this study, and the DAMs of *C. sinensis* before and after air drying were measured. Finally, the response of *C. sinensis* to air drying was determined to provide a theoretical basis for its medicinal research and guide consumers to choose the *C. sinensis* corresponding to their needs.

## 2. Materials and Methods

### 2.1. Sample Collection

**Figure 1:**
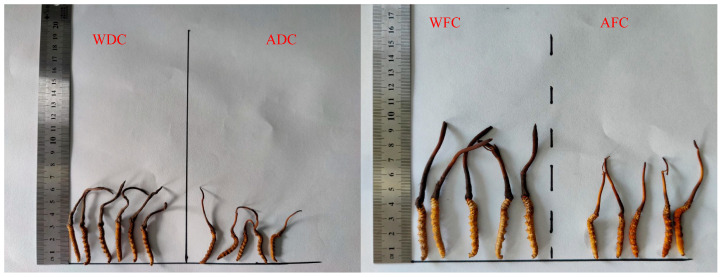
Four groups’ samples of *C. sinensis*. (WFC) wild fresh *C. sinensis*; (AFC) artificially fresh *C. sinensis*; (WDC) wildly dried *C. sinensis*; (ADC) artificially dried *C. sinensis*.

AFC (6 repeats) samples were purchased from Shenzhen Dongyangguang Industrial Development Co., Ltd. in Shenzhen China (https://www.dyg.cn/, accessed on 12 November 2023), and WFC (6 repeats) samples were purchased from Qinghai Qingqitang Trading Co., Ltd. (Xining, China), and were collected from Zaduo county, Yushu city, Qinghai province, China (95°38′12″ E, 33°8′15″ N, elevation: 4436 m). On 20 May 2023, six roots of each of the AFC (6 repeats) and WFC (6 repeats) were taken to be air dried at 22 °C while protected from light and without the application of additional treatments; they were weighed once every 6 h until the mass no longer changed significantly on two consecutive occasions. The moisture content was determined to be 13.23% in accordance with the standard of dried *C. sinensis*, and it was determined that the drying was completed to obtain 6 roots of ADC and WDC, respectively (Figure 1).

### 2.2. Sample Pretreatment

Four groups of *C. sinensis* samples were rinsed three times using sterile distilled water and then stroma and sclerotia mixture was ground in liquid nitrogen. A 50 mg sample was taken in a 2 mL EP tube and 250 mL of liquid extractant (methanol:water = 4:1) was added and used to present the metabolites. The sample was vortexed and shaken at −10 °C for 5 min (50 Hz) to allow the metabolites to be fully extracted and was then centrifuged at 4 °C, 15,000 rpm, for 20 min. The supernatant was collected through a 5 μm filter membrane to remove the larger particulate matter and microorganisms and transferred to the injection vials for LC-MS analysis, and 6 replicates each [25]. Aliquots of all samples were mixed to make quality control (QC) samples, processed as above, for testing the stability of the system and to ensure reliable data for the assay.

### 2.3. UPLC-MS/MS Analysis

The chromatographic column of Agilent 1290 Infinity LC (100 nm × 2.1 mm, 1.7 μm) was adopted. The UPLC conditions were column temperature 40 °C, flow rate 0.2 mL·min^−1^, mobile phase A (H_2_O:2 mM ammonium acetate:2 mM ammonia water = 5:47.5), and mobile phase B was acetonitrile. The chromatographic elution procedure in ESI positive mode was: 0–0.5 min, A:B = 5:95, 0.5–7 min, A:B = 35:65, 7–8 min, A:B = 60:40, 8–9 min, A:B = 5:95, 9–12 min, and B was maintained at 95%. The ESI negative mode is: 0–0.5 min, A:B = 5:95, 0.5–7 min, A:B = 10:90, 7–8 min, A:B = 35:65, 8–9 min, A:B = 5:95, 9–12 min, and B was maintained at 95%. Both A and B are linear changes [26].

The samples were kept at 4 °C throughout the injection process, and the stability of the system was tested and evaluated by inserting QC samples into the sample queue using continuous analysis with randomized sequential injection. The measured data were subjected to a simple screening of parameters such as retention time, mass-to-charge ratio, and so on. Then, peak extraction and peak area quantification were performed with a mass deviation of 5 ppm, a signal intensity deviation of 30%, a signal-to-noise ratio of 3, a minimum signal intensity of 100,000, summed ions, etc. The background ions were removed with blank samples, and the molecular ion peaks and fragment ions were compared with the mzCloud (https://www.mzcloud.org/, accessed on 12 November 2023), mzVault, and Masslist databases to predict the molecular formula, and the quantitative results were normalized to obtain the final identification and quantitative results of the data.

The ESI source conditions were set as follows: Ion Source Gas1 (Gas1) as 60, Ion Source Gas2 (Gas2) as 60, curtain gas (CUR) as 30, source temperature: 600 °C, IonSpray Voltage Floating (ISVF) ±5500 V. In MS only acquisition, the instrument was set to acquire over the *m*/*z* range 60–1000 Da, and the accumulation time for TOF MS scan was set at 0.20 s/spectra. In auto MS/MS acquisition, the instrument was set to acquire over the *m*/*z* range 25–1000 Da, and the accumulation time for product ion scan was set at 0.05 s/spectra. The product ion scan is acquired using information dependent acquisition (IDA) with high-sensitivity mode selected. The parameters were set as follows: the collision energy (CE) was fixed at 35 V with ±15 eV; declustering potential (DP), 60 V (+) and −60 V (−); exclude isotopes within 4 Da, candidate ions to monitor per cycle: 10.

### 2.4. Data Processing and Metabolite Identification

Data were collected and processed using MassLynx 4.1 for total peak area normalization. Multivariate statistical analyses of the data were performed using the R language ropls package, including principal component analysis (PCA), orthogonal partial least squares-discriminant analysis (OPLS-DA), and S-plot analysis of multiplicity of differences. Differential metabolite data were obtained by screening the minimum criteria for differential metabolite screening by simultaneously satisfying the following conditions: *p* ≤ 0.05, variable importance in projection (VIP) > 1, S-Plot > |0.8|. The relative quantitative hierarchical cluster plots of metabolites and differential metabolites were performed by the pheatmap program package in Rv 3.3.2. based on the data obtained by MS/MS in KEGG (https://www.genome.jp/kegg/pathway, accessed on 12 November 2023), Metlin (http://metlin.scripps.edu, accessed on 12 November 2023), and MoNA (https://mona.fiehnlab.ucdavis.edu, accessed on 12 November 2023) databases to further match the annotations and obtain accurate information regarding metabolites. Based on the MetPA analysis, the relative response values of metabolic pathways were obtained according to the relative response values of identified metabolites in metabolic pathways and the dimensionality reduction algorithm, from which the correlation coefficients between metabolic pathways were calculated and the metabolic pathway association network diagrams were drawn.

## 3. Results

### 3.1. Overview of Metabolite Profiling

**Figure 2:**
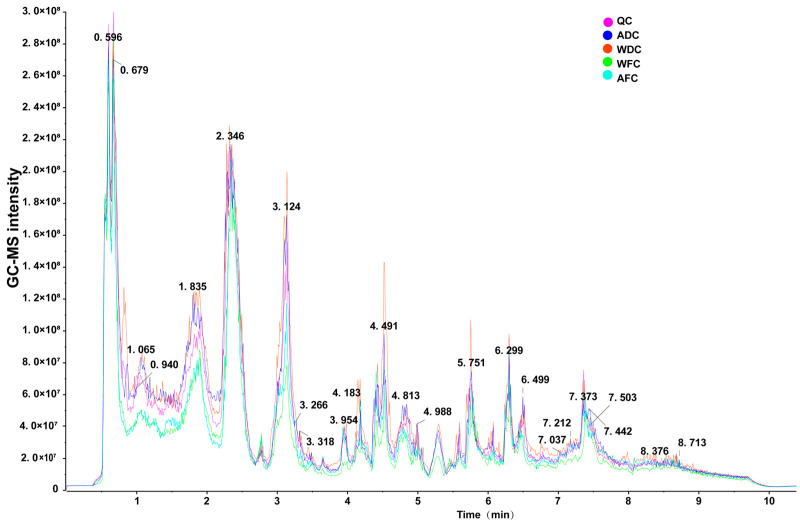
Total ion chromatograms of QC (quality control), ADC, WDC, WFC, and AFC non-targeted metabolomics methods. The numbers in the figure are retention times.

**Figure 3:**
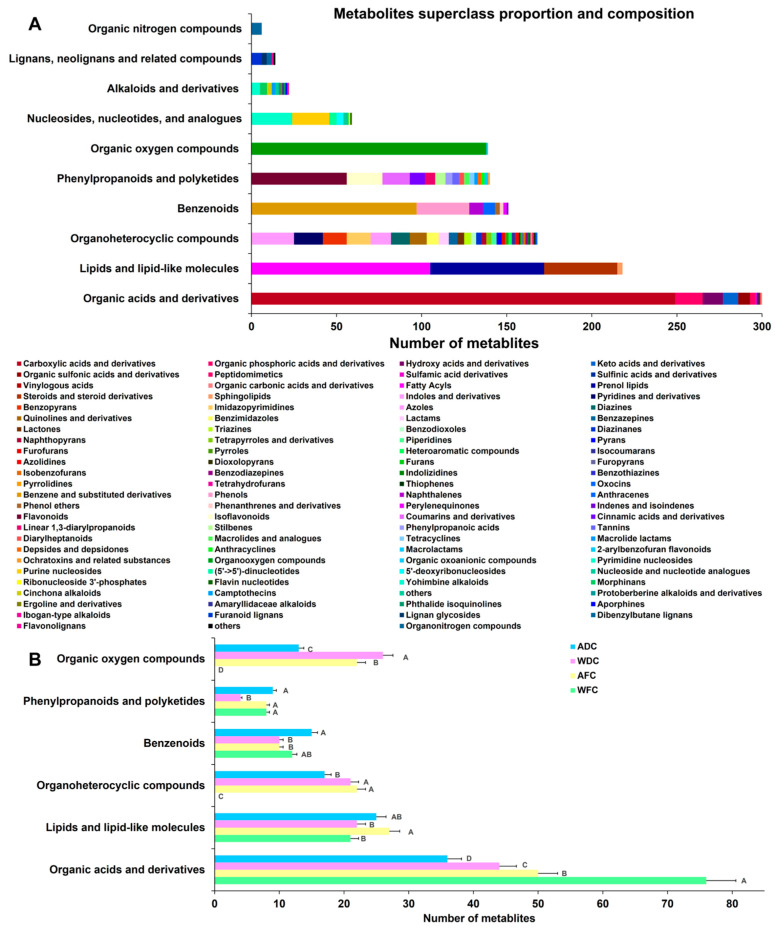
(**A**) Classification histogram of metabolites identified in the top 10 superclasses. (**B**) The expression of the top six superclasses in the four groups’ sample. Different capital letters in the same group showed significant differences at the level of *p* < 0.05.

In this study, 24 samples were studied, including AFC, WFC, ADC, and WDC. The GC-MS total ion current (TIC) chromatograms of four groups *C. sinensis* samples were obtained by non-targeted metabolomics detection (Figure 2). There were significant differences in the peak, position, and height between the four groups’ samples, focusing on the time of 0.5–7 min, and the differential metabolites of the four groups samples were observed. The metabolic profile showed that a total of 2638 metabolites (Table S1) were identified and annotated, which were divided into 18 superclasses and 38 classes (Table S2). The most abundant superclasses were organic acids and derivatives (300 metabolites, 24.4101%), followed by lipids and lipid-like molecules (218 metabolites, 17.7380%), organoheterocyclic compounds (168 metabolites, 13.6697%), benzenoids (151 metabolites, 12.2864%), phenylpropanoids and polyketides (140 metabolites, 11.3914%), and organic oxygen compounds (139 metabolites, 11.3100%) (Figure 3A). The most abundant classes were carboxylic acids and derivatives (249 metabolites, 20.2640%), followed by organooxygen compounds (138 metabolites, 11.2286%), fatty acyls (105 metabolites, 8.5435%), benzene and substituted derivatives (97 metabolites, 7.8926%), prenol lipids (67 metabolites, 5.4516%), flavonoids (56 metabolites, 4.5566%), steroids and steroid derivatives (43 metabolites, 3.4988%), phenols (31 metabolites, 2.5224%), indoles and derivatives (25 metabolites, 2.0342%), and pyrimidine nucleosides (24 metabolites, 1.9528%). This is consistent with the main metabolite types of fungi.

For the expression of the top six superclasses, differential expression analysis was performed in four groups samples (Figure 3B). The results showed that fresh *C. sinensis* contained more organic acids and derivatives and lower abundance of lipids and lipid-like molecules (*p* < 0.05) than dried *C. sinensis* (WFC vs. WDC, AFC vs. ADC). It is worth noting that WFC lacks organoheterocyclic compounds and organic oxygen compounds.

### 3.2. Multivariate Statistical Analysis

**Figure 4:**
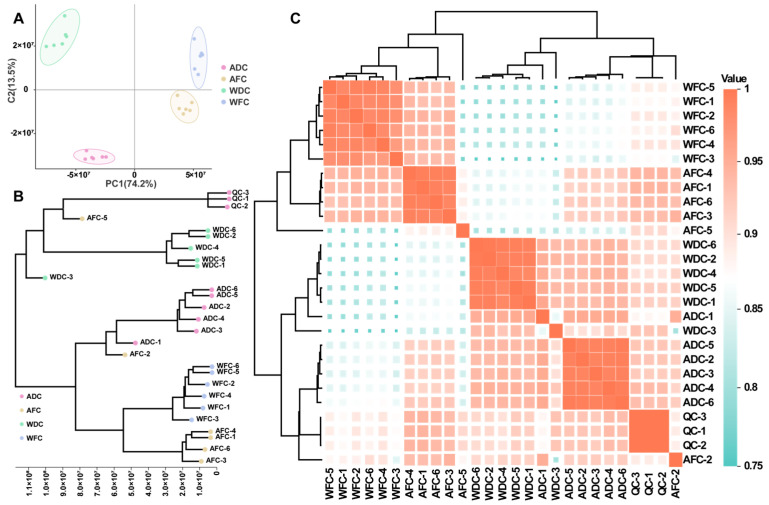
Multivariate statistical analysis of four groups samples. (**A**) PCA analysis of four groups samples. (**B**) Cluster analysis of four groups’ samples. (**C**) UMAP of four groups samples.

To determine the differences in metabolites among the four groups samples, a multivariate statistical analysis was performed using MS-normalized peak intensity as an indicator of metabolite abundance. Based on the obtained metabolite data set, the dimensionality reduction results of PCA (principal component analysis) showed that ADC, AFC, WDC, and WFC were significantly divided into four groups samples with a relatively long distance (Figure 4A), indicating that the metabolites of *C. sinensis* from different sources were significantly different. Air drying had a great effect on the metabolites of *C. sinensis*. Cluster analysis and UMAP (uniform manifold approximation and projection) found that ADC and WDC cluster together, and AFC and WFC cluster together (Figure 4B,C). This showed that the DAMs between fresh (WFC and AFC) and dried (WDC and ADC) *C. sinensis* were significant.

In PCA analysis, the first two PCs accounted for 87.7% of the total variance (PC1 = 74.2%, PC2 = 13.5%), indicating that the separation of metabolites from samples was clean, and the effect of principal component analysis was better. However, PCA analysis cannot remove intra-group errors and random errors, cannot overcome the integrity of the data, and cannot accurately distinguish between-group differences and differential metabolites between samples. OPLS-DA is a supervised discriminant analysis statistical method. By removing the data changes unrelated to the categorical variable Y in the independent variable X, the classification information is mainly concentrated on one principal component. In this study, OPLS-DA can better obtain the difference information between groups and predict the grouping of samples by establishing a model between metabolite expression and grouping relationship. In the OPLS-DA score plot, different species of *C. sinensis* were significantly distinguished (Figure S1A,B), which was consistent with the results of PCA. It was verified that the metabolites of the four groups samples were significantly different.

In the OPLS-DA score plot, fresh and dried *C. sinensis* were significantly distinguished, indicating that there were significant differences in the metabolic profiles of fresh and dried *C. sinensis*. The OPLS-DA model had high interpretability ($R_{X(WFC\;vs.\;WDC)}^{2}$ = 0.832, $R_{Y\left(WFC\;vs.\;WDC\right)}^{2}$ = 1, $R_{X(AFC\;vs.\;ADC)}^{2}$ = 0.811, and $R_{Y(AFC\;vs.\;ADC)}^{2}$ = 0.997) and predictability ($Q_{WFC\;vs.\;WDC}^{2}$ = 0.997, and $Q_{AFC\;vs.\;ADC}^{2}$ = 0.997). After 200 permutation tests (Figure S1C,D), it was found that all green Q^2^ values on the left side were less than the yellow R^2^ values, and the validation intercepts of R^2^ and Q^2^ were $R_{WFC\;vs.\;AFC}^{2}$ = 0.86, $Q_{WFC\;vs.\;AFC}^{2}$ = −0.15, $R_{WDC\;vs.\;ADC}^{2}$ = 0.82, and $Q_{WDC\;vs.\;ADC}^{2}$ = −0.08, respectively, indicating that the model was not overfitted and the results were reliable. Compared with WDC, WFC has more abundant Lpc 18:2, Acetyl coenzyme a, Glycerophosphocholine, Medermycin, N-n’-diphenyl-p-phenylenediamine, Ng, ng-dimethyl-l-arginine, and Chlormadinone acetate. In WDC, Oleamide, L-arginine, Tyramine, Adenosine, Ser-leu-ile-gly-lys-val-amide, Dehydrophtosphingosine, and S-methyl-5′-thioadensine were more abundant (Figure S1E). AFC is rich in betaine, Lpc 18:2, Medermycin, L-carnitine, Fluvoxamine, Acetyl coenzyme a, and N-N′-diphenyl-p-phenylenediamine. In ADC, Chlormadinone acetate, Fenpropidin, Glycerophosphocholine, S-methyl-5′-thioadensine, Oleamide, His-arg, Adenosine, Terfenadine, and Acetylcarnitine were detected more (Figure S1F).

The results showed that the abundance of Lpc 18:2, Acetyl coenzyme a, and N-N’-diphenyl-p-phenylenediamine in fresh *C. sinensis* was higher. Oleamide and Adenosine abundance was higher in dried *C. sinensis*. These DAMs may be involved in the air-drying process.

### 3.3. Screening of DAMs and Hierarchical Cluster Analysis (HCA)

**Figure 5:**
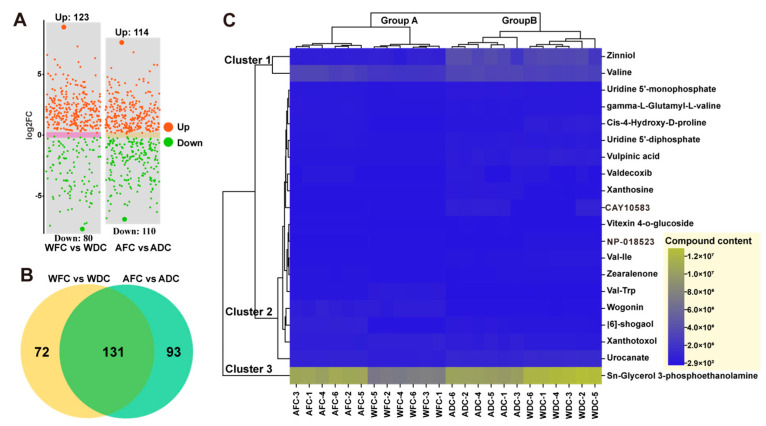
DAMs analysis of the four groups’ samples. (**A**) scatterplot of DAMs. (**B**) Numbers of upregulated and downregulated DAMs in four groups samples. (**C**) Dendrogram and heatmap of four groups samples (*C. sinensis*) from different origins based on top 20 differential metabolites content.

The screening conditions for significant DAMs were VIP ≥ 1 and *T*-test *p* < 0.5. To visualize the similarities and differences in metabolites in four groups samples, scatter plots and Venn diagrams of DAMs were used to describe them in detail (Figure 5A,B). By comparing DAMs in fresh and air-dried *C. sinensis* samples, a total of 427 DAMs (Tables S3 and S4) were detected, 237 were upregulated and 190 were downregulated. Among them, organic acids and derivatives, and lipids and lipid-like molecules are the main superclasses. The contents of fatty acids and conjugates, linolenic acids and derivatives, and amino acids, peptides, and analogues in fresh *C. sinensis* samples were higher than those in dried *C. sinensis* samples, such as Oleic acid, Linoleic acid, Phenylalanine, etc. On the other hand, levels of carbohydrates and carbohydrate conjugates, pyrimidine nucleosides, and cinchona alkaloids decreased. For example, Hydroquinidine, Uridine, Uracild, etc. (Tables S5 and S6).

This indicated that the air-drying process led to dramatic changes in the metabolites of fresh *C. sinensis*. Such changes may lead to changes in the pharmacological effects of fresh and dried *C. sinensis*. This study reported the types and quantities of metabolic variations, which will contribute to the further study of its pharmacological functions.

Based on the top 20 metabolites, the HCA of four groups sample metabolites was carried out. Different metabolites were clustered into one class, which showed that the expression patterns of different samples were closer. The clustering results of the differences showed that the expression of different samples was different among the samples. The four groups’ samples were clustered into two groups. Group A is AFC and WFC, and group B is ADC and WDC, which is consistent with the results of previous sample clustering and UMAP analysis (Figure 4B,C). This indicated that there were significant differences in metabolites between fresh samples and air-dried samples, but the expression of metabolites in each group was similar. At the same time, it can be divided into three clusters according to the content of metabolites in the sample (Figure 5C). Cluster 1 had higher metabolite content, such as Zinniol and Valine, cluster 2 had lower overall content, and cluster 3 had only Sn-glycerol-3-phosphethanolamine, but its content was the highest. To further compare the differences in the content of these metabolites, it is necessary to analyze them quantitatively.

The results showed that the abundances of Valine, Zinniol, Lysine, Cay10583, Vulpinic acid, Uridine 5’-diphosphate, Uridine 5’-monophosphate, and Val-ile were significantly higher in dried *C. sinensis* than in fresh *C. sinensis* (*p* < 0.05) (Table 1). This suggests that drying increases the levels of these metabolites. In contrast, the contents of Xanthotoxol, Vitexin-4-o-glucoside, Val-trp, and Wogonin were higher in fresh *C. sinensis*. This shows that these metabolites are destroyed by air drying.

### 3.4. KEGG Annotation and Metabolite Enrichment Analysis

**Figure 6:**
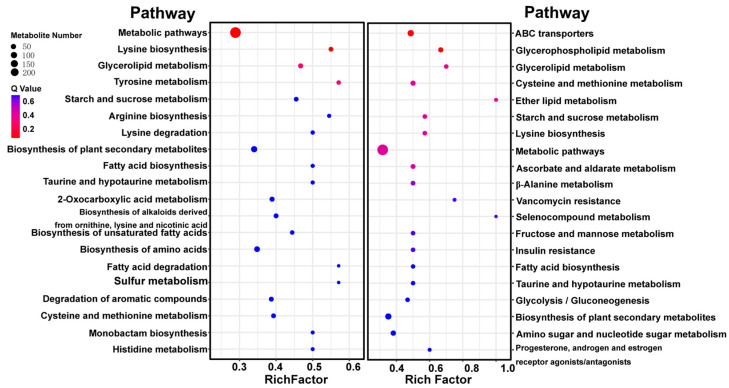
Differentially expressed metabolic pathway (top 20) in the four groups’ samples of *C. sinensis*. On the left is WFC vs. ADC and on the right is AFC vs. ADC.

**Figure 7:**
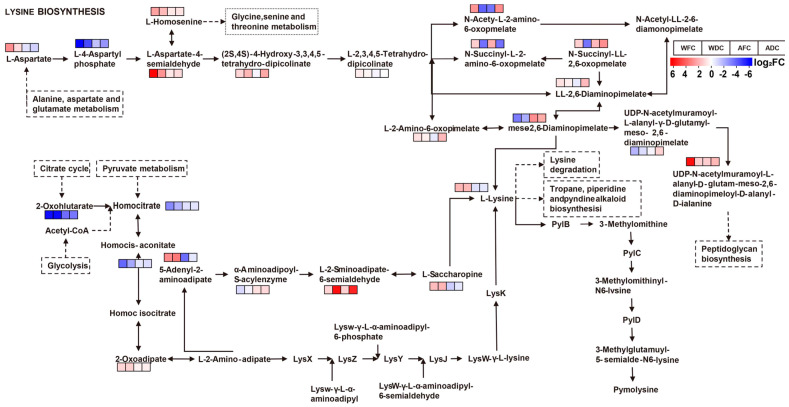
KEGG maps of key DAMs in the four groups’ samples of *C. sinensis*. The colored box in front of each metabolite indicates the corresponding log_2_ FC value. The dashed boxes indicate different metabolic pathways.

KEGG is a database that contains rich information about metabolite pathways and interactions. The pathway information may explain the biochemical function of compounds that can be activated by different metabolites. The first 20 enriched KEGG results showed that the DAMs contained in metabolic pathways were the most abundant in WFC vs. WDC and AFC vs. ADC (Figure 6). In addition, biosynthesis of plant secondary metabolites, lysine biosynthesis, glycerolipid metabolism, cysteine and methionine metabolism, fatty acid biosynthesis, starch and sucrose metabolism, and taurine and hypotaurine metabolism are common enrichment pathways. This suggests that these pathways are highly responsive metabolic pathways in air-dried samples and that lysine biosynthesis is one of the most significant pathways. The author constructed Figure 7 with reference to the pathway of lysine biosynthesis in KEGG (Figure S2). Among them, blue and red highlight the metabolites annotated by KEGG. The results showed that after air drying, the citrate cycle and pyruvate metabolism entered lysine biosynthesis through 2-oxoglutarate and Homocitrate, respectively, resulting in a significant increase in L-saccharopine and L-lysine content. Alanine, aspartate, and glutamate metabolism synthesize more L-aspartate to promote the synthesis of L-lysine. The high content of L-lysine entered lysine degradation and pyrrolysine to resist air drying.

## 4. Discussion

*C. sinensis* is widely loved by the public and is of interest to pharmacologists because of its diversity of metabolites and high medicinal value. However, scholars have focused most of their attention on fresh *C. sinensis* and have not performed much research on dried *C. sinensis*. The original source of Chinese medicinal materials is dried *C. sinensis*. Therefore, a comparative analysis of the metabolites of fresh and dried *C. sinensis* was conducted to clarify their metabolic characteristics and to speculate on their possible medicinal value and to ultimately meet the needs of the public. The results showed that fresh *C. sinensis* was richer in organic acids and derivatives while the abundance of lipids and lipid-like molecules was higher in dried *C. sinensis*. There was no significant difference in benzenoid, phenylpropanoid, or polyketide contents. It should be noted that dried *C. sinensis* may either lack or contain only a small amount of organic heterocyclic compounds, organic oxygen compounds, or other metabolites.

It has been reported that the loss of volatile compounds, such as organic acids and their derivatives, organic heterocyclic compounds and organic oxygen compounds (which are prone to decomposition reactions), phenols, acids, and alkanes, exacerbates the decrease in their abundance in dry environments [27,28]. In contrast, lipids and lipid-like molecules, due to their stable chemical structure, undergo only slow oxidation during the drying process to form short-chain acids, short-chain aldehydes, and short-chain ketones, maintaining a high abundance while promoting amino acid metabolism and carbohydrate metabolism [29,30,31]. Finally, there was a decrease in the abundance of amino acids, peptides, and analogues and an increase in the abundance of fatty acids and conjugates as well as lineolic acids and their derivatives [32,33]. Finally, Valine, Zinniol, Lysine, Cay10583, Vulpinic acid, Uridine 5’-diphosphate, Uridine 5’-monophosphate, and Valile were enriched in dried *C. sinensis*. The abundance of Xanthotoxol, Vitexin-4-o-glucoside, Val-trp, and Wogonin decreased. Strong lipid oxidation occurs in frozen (−20 °C) fresh *C. sinensis* [34], which promotes the synthesis of various amino acids, aldehydes, and ketones and releases energy to resist low-temperature environments. The drying process may not activate this process, and the abundance is relatively stable [35]. In addition, it has also been reported that amino acids and polysaccharides are stress-responsive substances, such as proline, arginine, citrulline, and tryptophan [36,37,38,39,40], which can significantly improve the individual’s ability to resist stress [41,42].

These studies have indicated that amino acid metabolism is active in the process of adversity, including but not limited to citrulline metabolism, arginine metabolism, histidine metabolism, tryptophan metabolism, glycine metabolism, and proline metabolism [43,44,45,46,47], resulting in dramatic changes in metabolites. In addition, this study also found that the content of valine and lysine in dried *C. sinensis* was higher, and more importantly, lysine biosynthesis was one of the significant enrichment pathways for drying.

It has been shown that lysine can improve the oxygen free-radical scavenging ability of individuals, inhibit the increase in reactive oxygen species and its possible subsequent damage, and can serve as an essential antioxidant [48,49]. It can also fight viral pathogens by destroying cell membrane structure and can improve T-cell lymphocytes to maintain non-innate immune activity, with strong antibacterial activity and the ability to regulate individual immunity [50]. In vitro mouse models have also demonstrated anti-apoptotic effects in lysine, reducing apoptosis of primary cardiomyocytes by eliminating the harmful effects of H_2_O_2_ [51]. All the above studies have demonstrated that lysine has the pharmacological effect of improving individual immune capacity and treating heart failure, while dried *C. sinensis* has higher lysine abundance and lysine biosynthesis intensity. Therefore, when the above symptoms occur, drying *C. sinensis* is recommended first.

Whether it is species or content, there are considerable differences in metabolites between fresh and dried *C. sinensis*, resulting in differences in their medicinal functions. Adenosine, cordycepin, cordycepin acid, and polysaccharides are generally considered to be the main medicinal components of *C. sinensis* [52]. Among them, adenosine content was used as a biomarker to evaluate the quality of *C. sinensis*. Adenosine can effectively inhibit the excessive excitation of neurons in the central nervous system and play an anticonvulsant role by inhibiting the release of synaptic front-end neurotransmitters [53]. The molecular structure of 3’-deoxyadenosine is CsHyON, which is essentially one of the derivatives of adenosine and belongs to nucleosides, nucleotides, and analogues [54]. Studies have confirmed that nucleosides, nucleotides, and analogues have strong bactericidal, antiviral, and anticancer potential [55]. This study found that there was no significant difference between fresh and dried *C. sinensis* (Tables S5 and S6). Therefore, both fresh and wild *C. sinensis* may be selected for the of viral diseases or cancer. For the sake of economic cost, fresh *C. sinensis* is recommended.

In summary, air drying significantly changed the metabolic profile of *C. sinensis* by enhancing lysine biosynthesis, resulting in different pharmacological effects and medicinal values of fresh and air-dried *C. sinensis*. This study analyzed these metabolites and possible medicinal differences in detail, providing scientific guidance for the public’s choice of medicine and health care.

## 5. Conclusions

In this study, we compared the metabolic profiles of dried and fresh samples of wild and artificial *C. sinensis* and found differences in the metabolites of fresh and air-dried *C. sinensis*. Air drying significantly altered the composition and content of *C. sinensis*, which was mainly characterized by higher abundance of organic acids and derivative and lower abundance of lipids and lipid-like molecules in fresh *C. sinensis*. A total of 427 DAMs were identified, of which 237 were upregulated in expression, mainly in the following areas: carbohydrates and carbohydrate conjugates, pyrimidine nucleosides, and cinchona alkaloids. The expression of 190 DAMs was downregulated, mainly in fatty acids and conjugates, lineolic acids and derivatives, amino acids, peptides, and analogues. HCA and quantitative analyses showed that air drying increased levels of Valine, Zinniol, Urocanate, Vulpinic acid, and Uridine 5’-diphosphate and decreased levels of Xanthotoxol, Vitexin-4-o-glucoside, Val-trp, and Wogonin. These DAMs were also shown to be potential biomarkers for *C. sinensis*. KEGG enrichment analysis revealed that lysine biosynthesis was the most significantly enriched pathway. Annotation of these DAMs to lysine biosynthesis revealed that citrate cycle and pyruvate metabolism enter lysine biosynthesis via 2-oxohlutarate and Homocitrate, respectively, resulting in significantly higher L-saccharopine and L-lysine content. Alanine, Aspartate, and Glutamate metabolism synthesized more L-aspartate to promote L-lysine synthesis. Thus, high levels of L-lysine go into lysine degradation, and pymolysine is thus the most active metabolic pathway during the drying of fresh *C. sinensis* and indirectly leads to differences in metabolic profiles.

## Figures and Tables

**Table 1:** The relative abundance of metabolite top 20 was differentially expressed in four groups samples of *C. sinensis*. Capital letters indicate a significant difference at the *p* < 0.05 level.

Compound	ADC (Relative Abundance)	AFC (Relative Abundance)	WDC (Relative Abundance)	WFC (Relative Abundance)
Sn-Glycerol 3-phosphoethanolamine	1.06 × 10^7^ ± 9.48 × 10^4 C^	1.11 × 10^7^ ± 9.17 × 10^4 B^	1.28 × 10^7^ ± 1.43 × 10^5 A^	7.73 × 10^6^ ± 9.88 × 10^4 D^
Valine	3.61 × 10^6^ ± 2.86 × 10^4 A^	3.34 × 10^6^ ± 1.22 × 10^5 B^	3.26 × 10^6^ ± 3.88 × 10^4 B^	2.34 × 10^6^ ± 3.32 × 10^4 C^
Zinniol	3.72 × 10^6^ ± 3.04 × 10^5 A^	7.05 × 10^5^ ± 2.70 × 10^4 B^	3.59 × 10^6^ ± 2.18 × 10^5 A^	8.88 × 10^5^ ± 7.58 × 10^4 B^
Xanthotoxol	6.65 × 10^5^ ± 3.63 × 10^4 B^	6.70 × 10^5^ ± 8.54 × 10^4 B^	5.22 × 10^5^ ± 1.65 × 10^4 C^	7.80 × 10^5^ ± 3.40 × 10^4 A^
NP-018523	1.20 × 10^4^ ± 7.87 × 10^2 C^	8.15 × 10^2^ ± 1.26 × 10^2 D^	1.52 × 10^5^ ± 6.86 × 10^3 A^	7.60 × 10^4^ ± 2.56 × 10^3 B^
Urocanate	1.21 × 10^6^ ± 2018 × 10^4 B^	9.68 × 10^5^ ± 1.20 × 10^4 C^	1.45 × 10^6^ ± 1.40 × 10^4 A^	6.61 × 10^5^ ± 9.16 × 10^3 D^
CAY-10583	6.01 × 10^5^ ± 1.20 × 10^5 A^	2.00 × 10^4^ ± 1.47 × 10^3 C^	3.23 × 10^5^ ± 2.01 × 10^5 AB^	7.28 × 10^4^ ± 4.19 × 10^3 B^
Cis-4-Hydroxy-D-proline	2.37 × 10^5^ ± 2.32 × 10^3 C^	2.96 × 10^5^ ± 2.72 × 10^3 B^	6.56 × 10^5^ ± 1.52 × 10^4 A^	7.11 × 10^4^ ± 8.93 × 10^2 D^
Vulpinic acid	5.63 × 10^5^ ± 3.23 × 10^4 B^	1.41 × 10^5^ ± 1.41 × 10^3 C^	7.13 × 10^5^ ± 4.46 × 10^4 A^	1.77 × 10^5^ ± 4.91 × 10^3 C^
Uridine 5’-diphosphate	5.09 × 10^5^ ± 8.44 × 10^3 A^	2.78 × 10^5^ ± 8.31 × 10^3 B^	4.93 × 10^5^ ± 1.72 × 10^4 A^	1.52 × 10^5^ ± 5.17 × 10^3 C^
[6]-shogaol	3.85 × 10^5^ ± 3.28 × 10^4 B^	8.80 × 10^5^ ± 1.48 × 10^4 A^	2.35 × 10^5^ ± 1.07 × 10^4 C^	9.03 × 10^4^ ± 6.88 × 10^3 D^
γ-L-Glutamyl-L-valine	2.82 × 10^5^ ± 8.87 × 10^3 C^	3.96 × 10^5^ ± 1.77 × 10^4 A^	3.38 × 10^5^ ± 1.62 × 10^4 B^	1.72 × 10^5^ ± 7.52 × 10^3 D^
Uridine 5’-monophosphate	4.39 × 10^5^ ± 5.62 × 10^3 A^	3.69 × 10^5^ ± 2.87 × 10^3 B^	3.67 × 10^5^ ± 5.16 × 10^3 B^	2.04 × 10^5^ ± 1.84 × 10^3 C^
Xanthosine	4.60 × 10^5^ ± 1.82 × 10^4 A^	8.98 × 10^4^ ± 1.27 × 10^4 B^	5.87 × 10^4^ ± 3.18 × 10^3 C^	6.85 × 10^4^ ± 5.10 × 10^3 BC^
Zearalenone	1.09 × 10^5^ ± 1.39 × 10^4 B^	1.68 × 10^5^ ± 1.60 × 10^4 A^	1.71 × 10^5^ ± 2.20 × 10^3 A^	7.05 × 10^4^ ± 3.76 × 10^3 C^
Val-Ile	1.29 × 10^5^ ± 3.11 × 10^3 B^	6.93 × 10^4^ ± 2.17 × 10^3 D^	2.75 × 10^5^ ± 6.30 × 10^3 A^	1.06 × 10^5^ ± 2.81 × 10^3 C^
Vitexin 4-o-glucoside	1.89 × 10^4^ ± 9.78 × 10^3 B^	9.92 × 10^4^ ± 9.71 × 10^2 A^	1.33 × 10^4^ ± 5.41 × 10^3 B^	1.38 × 10^4^ ± 3.66 × 10^2 B^
Val-Trp	9.89 × 10^3^ ± 1.83 × 10^3 C^	1.54 × 10^5^ ± 9.06 × 10^3 B^	3.16 × 10^4^ ± 3.49 × 10^3 C^	5.17 × 10^5^ ± 1.01 × 10^4 A^
Wogonin	7.53 × 10^4^ ± 6.10 × 10^3 B^	6.88 × 10^5^ ± 5.49 × 10^4 A^	6.62 × 10^4^ ± 8.28 × 10^3 B^	6.12 × 10^5^ ± 2.39 × 10^4 A^
Valdecoxib	3.45 × 10^5^ ± 1.09 × 10^5 A^	2.00 × 10^5^ ± 6.26 × 10^4 AB^	1.97 × 10^5^ ± 8.74 × 10^4 AB^	1.15 × 10^4^ ± 3.63 × 10^2 B^

## Data Availability

Data are contained within the article and Supplementary Materials.

## Supplementary Materials

The following supporting information can be downloaded at: https://www.mdpi.com/article/10.3390/foods13010048/s1, Figure S1: (A,B) OPLS-DA score plot and ellipses represent the 95% confidence region for Hoteling’s T^2^. (C,D) Statistical validation of the OPLS-DA model using 200 permutation analysis. (E,F) Corresponding S-plot of OPLS-DA. The green value represents the VIP ≥ 1. (A,C,E): WFC vs. WDC; (B,D,F): AFC vs. ADC; Figure S2: Kegg pathway of lysine biosynthesis; Table S1: Detailed information of metabolites detected in ADCs, AFCs, WDCs, and WFCs; Table S2: Detailed classification information of metabolites detected in ADCs, AFCs, WDCs, and WFCs; Table S3: Details of the differential metabolites detected between WFCs and WDCs; Table S4: Details of the differential metabolites detected between AFCs and ADCs; Table S5: Differential metabolite classification information between WFCs and WDCs; Table S6: Differential metabolite classification information between AFCs and ADCs.
