# Supplementary figures and images for: Effect of Air Drying on the Metabolic Profile of Fresh Wild and Artificial Cordyceps sinensis
Source: Foods. 2023 Dec 21;13(1):48. doi: 10.3390/foods13010048 (PMC10778509; doi:10.3390/foods13010048)

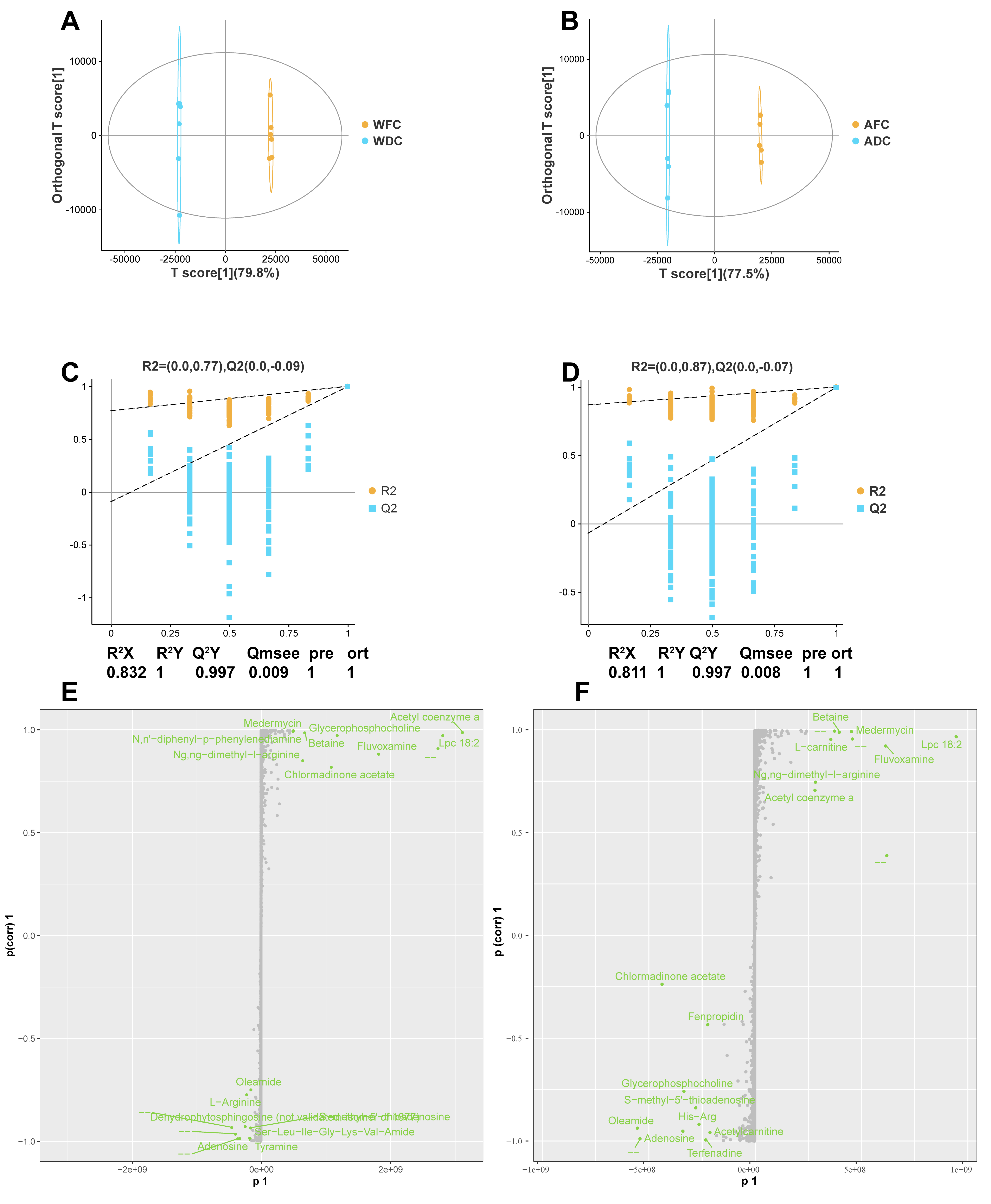

Supplement: Supplementary file 1 [file foods-13-00048-s001.zip › Figure S1. Multivariate statistical analysis of four samples.jpg]

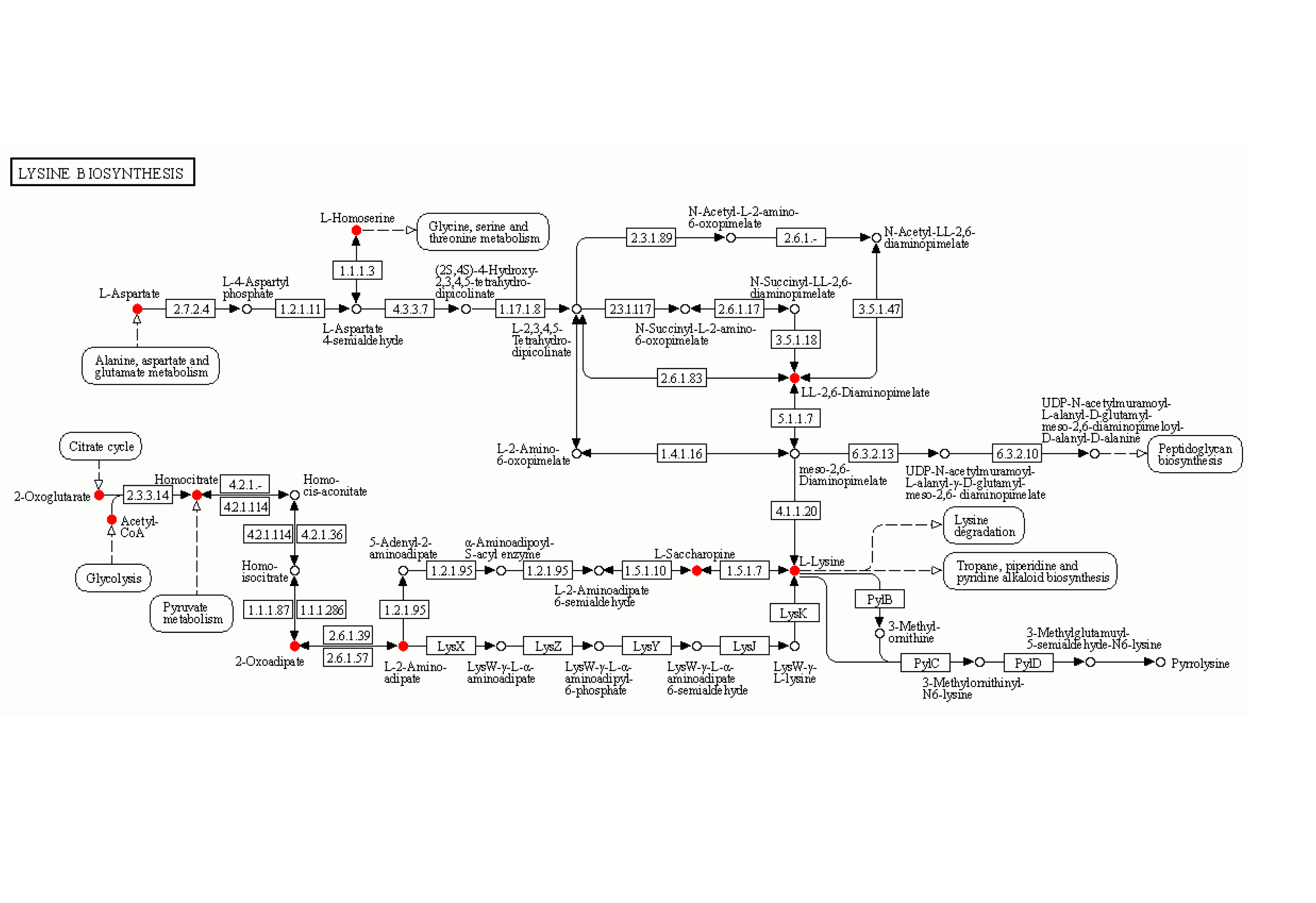

Supplement: Supplementary file 1 [file foods-13-00048-s001.zip › Figure S2. Kegg pathway-lysine biosynthesis.jpg]
